# Dental students’ awareness and attitudes toward HPV-related oral cancer: a cross sectional study at the University of Jordan

**DOI:** 10.1186/s12903-019-0864-8

**Published:** 2019-08-01

**Authors:** Malik Sallam, Esraa Al-Fraihat, Deema Dababseh, Alaa’ Yaseen, Duaa Taim, Seraj Zabadi, Ahmad A. Hamdan, Yazan Hassona, Azmi Mahafzah, Gülşen Özkaya Şahin

**Affiliations:** 1 Department of Pathology, Microbiology and Forensic Medicine, School of Medicine, the University of Jordan, Amman, Jordan; 2 Department of Clinical Laboratories and Forensic Medicine, Jordan University Hospital, Queen Rania Al-Abdullah Street-Aljubeiha/P.O. Box: (13046), Amman, Jordan; 3 Department of Translational Medicine, Faculty of Medicine, Lund University, Malmö, Sweden; 4 School of Dentistry, The University of Jordan, Amman, Jordan; 5 Department of Oral and Maxillofacial Surgery, Oral Medicine and Periodontology, Jordan University Hospital, Amman, Jordan; 6 Department of Clinical Microbiology, Laboratory Medicine, Skåne University Hospital, Lund, Sweden

**Keywords:** Education, Tumor, Attitude, HPV-16, Squamous cell carcinoma, Early detection, Oral cavity cancer, Vaccine

## Abstract

**Background:**

The incidence of human papilloma virus (HPV)-related oral cancer has recently increased worldwide. The role of dentists is of prime importance in the early detection of oral cancer which would result in a favourable outcome for the patients. The aim of the current study was to assess the knowledge, awareness and attitudes of dental students, interns and postgraduate maxillofacial residents at the University of Jordan (UJ) to different aspects of oral cancer, particularly those related to HPV.

**Methods:**

A paper-based survey was conducted at UJ among all pre-clinical dental students (pre-clinical group), clinical dental students, interns and postgraduate maxillofacial residents (clinical group). The survey included five sections comprising 29 items. The sections included questions investigating oral cancer knowledge, oral cancer screening, HPV knowledge and the ability to discuss personal topics with patients.

**Results:**

A total of 376 respondents out of 1052 potential participants completed at least one item of the survey (study coverage of 35.7%). Among the study participants, the pre-clinical group represented 41.2% (*n* = 155) and the clinical group represented 58.8% (*n* = 221). The majority of participants in the clinical group showed better knowledge on oral cancer potential anatomic sites, clinical presentation and possible risk factors compared to the pre-clinical group. Most participants in the clinical group (*n* = 195, 88.2%) correctly identified HPV as a risk factor for oral cancer development. The majority of participants in the clinical group displayed suitable attitude towards oral cancer screening despite their desire for a reliable screening device and additional training in oral cancer screening. A number of limitations in basic knowledge about HPV was noticed among participants in the clinical group particularly related to unawareness of the vaccine availability. The majority of participants in the clinical group displayed hesitancy in discussing personal topics with the patients, including the history of previous sexually transmitted infections and sexual abuse.

**Conclusions:**

Gaps in knowledge regarding HPV-related oral cancer has been detected which necessitate intervention measures including curricular changes, training workshops and awareness campaigns.

**Electronic supplementary material:**

The online version of this article (10.1186/s12903-019-0864-8) contains supplementary material, which is available to authorized users.

## Background

Cancers of the oral cavity, oropharynx and lips represent a growing problem worldwide with an estimated incidence of about 448,000 cases and 228,000 deaths in 2018 [1]. Although the majority of cancers arising from these three sub-sites are squamous cell carcinomas, they have different major etiologic factors (ultra-violet exposure for lip cancer, tobacco, alcohol, and areca-nut chewing for oral cavity cancer, and human papilloma virus [HPV] infection for oropharyngeal cancer) [2, 3]. Furthermore, cancers arising from different anatomic sub-sites exhibit different biologic behaviour, and different prognosis and management [4–6].

The high mortality rate associated with oral cancer is related to late presentation of a large proportion of patients with advanced disease [7]. Thus, early diagnosis appears to be of prime importance for achieving a favourable outcome in the patients [8]. Although the oral cavity represents an easily accessible site for clinical examination, the lack of awareness in both the patients and health-care professionals precludes early detection of precancerous and early cancer lesions [9–11]. A promising strategy to improve survival among patients with oral cancer is increasing awareness and knowledge among the patients and health-care professionals [12]. Dentists represent a significant sector which faces this problem, thus focusing on this group with educational material is of high value [12]. Identifying the gaps in dental professionals’ knowledge and increasing their confidence in discussing HPV as a sexually transmitted infection (STI) is important to detect early cases [13].

The potential role of high-risk HPV types, particularly HPV-16 as risk factors for the development of oral cancer has been elucidated for decades [14, 15]. Additionally, the burden of HPV-related oral cancer is increasing worldwide [16, 17]. Various studies from developed countries (e.g. North America, Europe, Japan and Australia) reported that 17–56% of all oral cancers are HPV-related [18]. However, limited data are available from the less developed regions including the Middle East and North Africa (MENA) [18]. HPV infection with malignant strains is likely to play a role in oral cancer in the MENA despite the scarcity of data on this subject including a few studies from Yemen, Sudan, Syria, and Iran [19–23].

Several studies examined knowledge and awareness of patients, students, and dental professionals toward oral cancer in different parts of the world and reported variable results [10, 24–26]. Nevertheless, few studies examined knowledge of health care professionals about HPV-related oral cancer and their attitude toward HPV screening and discussing personal topics with patients [27, 28].

Earlier studies from Jordan aimed to assess general knowledge of oral cancer of the dental students, recently graduated medical and dental professionals, and among the patients [10, 29, 30]. However, no previous reports have been found in Jordan that assessed knowledge of HPV-related oral cancer among dental students. Thus, the objectives of the current project were: (1) to assess the general knowledge of dental students at the University of Jordan (UJ) regarding different aspects of oral cancer (anatomic sites, clinical presentation and risk factors). (2) to evaluate the attitude of clinical students regarding screening of oral cancer. (3) to assess the knowledge of dental students at UJ regarding different aspects of HPV infection. (4) to assess the attitude of the clinical students regarding the discussion of personal topics with patients.

## Methods

### Study participants

This cross-sectional study was conducted using a paper-based questionnaire that was distributed among pre-clinical doctor of dental surgery (DDS) students (2nd and 3rd year students), clinical DDS students (4th and 5th year students), interns, and postgraduate maxillofacial residents at the University of Jordan (UJ) and Jordan University Hospital (JUH). The survey was distributed among potential participants during December 2018 and January 2019. In subsequent analysis, the pre-clinical students were considered as one group “pre-clinical” and the 4th year, 5th year DDS students, interns and postgraduate residents were considered as a second group “clinical”.

At the time of manuscript writing, the DDS program at UJ comprised 196 credit hours distributed over five years. The curriculum first year entails basic science courses and elective courses and is considered a pre-med foundation year with candidates divided into the doctor of medicine program and into the DDS program. Pre-clinical courses taken during the second and third curriculum years include basic medical and dental sciences. The start of clinical courses is from the summer semester of the third year. Internship is a one-year period and postgraduate maxillofacial residency training entails clinical rotations for four years and the residents are also considered as postgraduate students.

The target study population was all DDS students from 2nd year up till postgraduate residents with the aim of reducing coverage error of the survey. The total number of potential study participants was 1052, distributed as follows: 2nd year (*n* = 337), 3rd year (*n* = 257), 4th year (*n* = 199), 5th year (*n* = 202), interns (*n* = 45) and postgraduate maxillofacial residents (*n* = 12).

### Ethical permission

The study was approved by the JUH institutional review board (IRB/296/2018) in accordance with the Declaration of Helsinki. Participation in the study was voluntary and anonymous. An informed consent was taken from every participant verbally, and the collected data were treated confidentially.

### Data collection

The survey items were adopted from previous studies among dentists and dental students in USA, Netherlands and Spain [26, 31, 32]. To assess the time needed to complete the survey, question wording, survey flow, and choice of response, a pilot test was performed on randomly selected participants from the pre-clinical and clinical groups (*n* = 10). Electronic-based survey was sent by e-mail (*n* = 5) and the paper-based survey (n = 5) was distributed in-person. No response was obtained from the five participants who received the survey by e-mail as compared to 100% response rate in the paper-based pilot survey. Hence, it was decided to conduct this study using the paper-based format. English language was used to conduct the survey as English is the official teaching language of dentistry at UJ. Minor modifications were made according to the results of pilot test, and the results of this pilot testing were not included in the final analysis. The final version of the survey took approximately 5–10 min to complete. Surveys were distributed at the beginning or the end of classes at lecture rooms for the DDS students. For interns and postgraduate residents, participation was offered in-person in the Department of Dentistry, JUH. In all cases, participation was voluntary and anonymous. The students were informed of the nature of the study through an information section at the start of the survey. Then, the self-administered survey was distributed and collected back after about 10 min.

### Survey items

The survey items were constructed in light of the study objectives that were mentioned previously in the introduction section. The survey comprised five sections, each of which consisted of several items with a total of 29 items in the entire questionnaire. The first section included questions on age, gender, nationality and current level of education. The second section included items related to oral cancer knowledge (anatomic sites, early signs, clinical presentation and risk factors). The third section included items related to attitude towards oral cancer screening. The fourth section was about HPV knowledge. The final section included items related to discussing personal topics with the patients (Additional file 1).

### Statistical analysis

Statistical analysis was conducted through IBM SPSS Statistics 22.0 for Windows. Two-sided Fisher’s exact test (FET) and Mann-Whitney *U* test (M-W) were used when appropriate. To compare the mean score for certain survey items across different educational levels, we used the Kruskal-Wallis (K-W) test. To compare the scores stratified by gender and nationality that were associated with being comfortable in asking the patients about personal topics, two-sided independent samples t-test was used. Statistical significance was considered for *p* < 0.050. To calculate the sample size margin of error, “Sample size calculator” available freely online from CheckMarket (“Sample size calculator”, CheckMarket, https://www.checkmarket.com/sample-size-calculator/. accessed 3 May 2019) was used.

## Results

### Study participants

A total of 376 participants out of 1052 potential participants completed at least one item of the survey yielding a study coverage of 35.7%. A hundred and fifty five participants were pre-clinical students (41.2%) and 221 participants were clinical students, interns and postgraduate residents (58.8%). The response was higher among the clinical group compared to the pre-clinical group (48.3% vs. 26.1%, *p* < 0.001, FET). The details of participants’ distribution were as follows: second-year pre-clinical students (*n* = 70, 18.6%), third-year pre-clinical students (*n* = 85, 22.6%), fourth-year clinical students (*n* = 124, 33%), fifth year clinical students (*n* = 67, 17.8%), interns (*n* = 24, 6.4%) and postgraduate residents (n = 6, 1.6%). The highest response rate was among the 4th year students (124/199, 62.3%), followed by the interns (24/45, 53.3%), postgraduate residents (6/12, 50.0%), 5th year students (67/202, 33.2%), 3rd year students (85/257, 33.1%) and 2nd year students (70/337, 20.8%). The calculated sample size margin of error was 4.06%, considering the 95% confidence level.

About three-fourths of the study population were females (*n* = 280), and the median age of the study participants was 21 years (mean: 21.2, interquartile range [IQR]: 20–22, Table 1). Gender-based comparison of age revealed no differences (median age 21 years for both, IQR: (20-22) vs. (20-23) for males and females, respectively, *p* = 0.168, M-W). Of the whole study sample, 57.7% of the students were Jordanians whereas 15.7% where of non-Jordanian citizenship (of 13 nationalities and 94.8% of non-Jordanian students came from the MENA countries). Data on nationality were missing for 100 participants (26.6%).

**Table 1:** Characteristics of the study participants

	Pre-clinical group^a^ N^b^ (%)	Clinical group^c^ N (%)	Total N (%)
	155 (41.2)	221 (58.8)	376
Median Age (Range)	20 (18–25)^d^	22 (20–33)^e^	21 (18–33)
Gender			
Male	42 (27.1)	53 (24.0)	95 (25.3)
Female	112 (72.3)	168 (76.0)	280 (74.5)
Data missing	1 (0.6)	0	1 (0.3)
Nationality			
Jordanian	84 (54.2)	133 (60.2)	217 (57.7)
Non-Jordanian^f^	20 (12.9)	39 (17.6)	59 (15.7)
Data missing	51 (32.9)	49 (22.2)	100 (26.6)

^a^Pre-clinical group: Pre-clinical doctor of dental surgery (DDS) students (2nd and 3rd year students), ^b^N: Number, ^c^Clinical group: Clinical DDS students (4th and 5th year students), interns, and postgraduate maxillofacial residents, ^d^Data on six participants were missing, ^e^Data on six participants were missing, ^f^Non-Jordanian: Countries of citizenship included Iraq, Kuwait, Palestine, Syria, Yemen, Saudi Arabia, Libya, Morocco, Algeria, USA, Canada and two different dual citizenship countries

### Oral cancer knowledge

All participants in the clinical group stated that they heard of oral cancer (*n* = 221) compared to 79.4% (*n* = 123) of the participants in the pre-clinical group (*p* < 0.001, FET, Table 2).

**Table 2:** Assessment of oral cancer knowledge and human papillomavirus (HPV) knowledge among study participants

Educational stage	Pre-clinical group^a^		Clinical group^b^		*P* value^c^
*Survey item*	YES N^d^ (%)	NO N (%)	YES N (%)	NO N (%)	
*Oral cancer Knowledge*					
Have you ever heard of oral cancer?	123 (79.4)	32 (20.6)	221 (100)	0	< 0.001
Where do you think oral cancer is found in the oral cavity?					
*Tongue*	49 (40.2)	73 (59.8)	214 (96.8)	7 (3.2)	< 0.001
*Lips*	29 (23.8)	93 (76.2)	128 (57.9)	93 (42.1)	< 0.001
*Palate*	58 (47.5)	64 (52.5)	134 (60.6)	87 (39.4)	0.023
*Jaw bone*	38 (31.1)	84 (68.9)	135 (61.1)	86 (38.9)	< 0.001
*Buccal mucosa*	72 (59.0)	50 (41.0)	143 (64.7)	78 (35.3)	0.351
*Floor of the mouth*	52 (42.6)	70 (57.4)	200 (90.5)	21 (9.5)	< 0.001
*Other sites*^*e*^	3 (2.5)	119 (97.5)	9 (4.1)	212 (95.9)	0.550
Which of the following describes the clinical appearance of the early lesion of oral cancer?					
*White lesion*	46 (37.7)	76 (62.3)	155 (70.1)	66 (29.9)	< 0.001
*Red lesion*	21 (17.2)	101 (82.8)	134 (60.6)	87 (39.4)	< 0.001
*Ulcer*	42 (34.4)	80 (65.6)	138 (62.4)	83 (37.6)	< 0.001
*Mass*	42 (34.4)	80 (65.6)	114 (51.6)	107 (48.4)	0.002
*Others*^*f*^	0	122 (100)	7 (3.2)	214 (96.8)	0.054
What are the signs and symptoms of oral cancer?					
*Mucosal bleeding*	44 (36.1)	78 (63.9)	111 (50.2)	110 (49.8)	0.013
*Difficulty in swallowing*	54 (44.3)	68 (55.7)	139 (62.9)	82 (37.1)	0.001
*Tooth mobility*	29 (23.8)	93 (76.2)	111 (50.2)	110 (49.8)	< 0.001
*Lymph node enlargement*	76 (62.3)	46 (37.7)	191 (86.4)	30 (13.6)	< 0.001
*Others*^*g*^	0	122 (100)	6 (2.7)	215 (97.3)	0.093
What are the risk factors for oral cancer?					
*Smoking*	102 (83.6)	20 (16.4)	216 (97.7)	5 (2.3)	< 0.001
*Alcohol*	38 (31.1)	84 (68.9)	194 (87.8)	27 (12.2)	< 0.001
*Positive family history*	51 (41.8)	71 (58.2)	168 (76.0)	53 (24.0)	< 0.001
*HPV*^*h*^	48 (39.3)	74 (60.7)	195 (88.2)	26 (11.8)	< 0.001
*HPV Knowledge*	Correct answer N (%)	Incorrect answer N (%)	Correct answer N (%)	Incorrect answer N (%)	*P* value
*-Have you ever heard of HPV?*	52 (65.0)	28 (35.0)	214 (100.0)	0	< 0.001
*-HPV causes AIDS* ^*i*^	28 (53.8)	24 (46.2)	154 (73.0)	57 (27.0)	0.011
*-HPV can cause a sexually transmitted infection*	43 (82.7)	9 (17.3)	194 (92.8)	15 (7.2)	0.032
*-Antibiotics can cure HPV infection*	40 (75.5)	13 (24.5)	186 (86.9)	28 (13.1)	0.054
*-Most HPV infections resolve within a short time*	16 (32.0)	34 (68.0)	46 (22.1)	162 (77.9)	0.145
*-Certain strains of HPV causes cervical cancer*	47 (88.7)	6 (11.3)	199 (94.8)	11 (5.2)	0.121
*-HPV causes herpes and cold sores*	13 (24.5)	40 (75.5)	120 (56.9)	91 (43.1)	< 0.001
*-A person can have HPV without knowing it*	40 (75.5)	13 (24.5)	192 (91.0)	19 (9.0)	0.004
*-HPV can cause oral cancer*	43 (82.7)	9 (17.3)	206 (97.2)	6 (2.8)	< 0.001
*-We have a vaccine for HPV*	22 (44.0)	28 (56.0)	78 (36.8)	134 (63.2)	0.419

^a^Pre-clinical group: Pre-clinical doctor of dental surgery (DDS) students (2nd and 3rd year students), ^b^Clinical group: Clinical DDS students (4th and 5th year students), interns, and postgraduate maxillofacial residents, ^c^*P* value: calculated using two-sided Fisher’s exact test. ^d^N: number. ^e^Other sites reported: salivary glands (n = 6), gingivae (n = 5), pharynx and larynx (n = 1). ^f^Others: painless mass (n = 5), non-healing lesion (n = 2). ^g^Others: painless mass (n = 5), tooth root resorption (n = 1). ^h^HPV: human papillomavirus. ^i^AIDS: acquired immune deficiency syndrome

Regarding the possible anatomic locations for oral cancer, the vast majority of the participants in the clinical group correctly identified the tongue and floor of the mouth (96.8 and 90.5% respectively). However, a considerable percentage of the participants in the clinical group did not recognize the following as potential sites for oral cancer: lips (42.1%), palate (39.4%), jaw bone (38.9%) and buccal mucosa (35.3%). Higher percentage of correct identification of oral cancer anatomic sites was observed in the clinical group compared to the pre-clinical group, except for the buccal mucosa (*p* = 0.351, Table 2, Additional file 2).

A considerable percentage of the participants in the clinical group did not recognize the following as possible oral cancer early signs: mass (48.4%), red lesion (39.4%), ulcer (37.6%) and white lesion (29.9%). However, the participants in the clinical group were more likely to correctly identify early signs of oral cancer compared to the pre-clinical group for all items (Table 2, Additional file 2).

For the clinical manifestations of oral cancer, the clinical group showed better knowledge in all items compared to the pre-clinical group and the majority correctly identified lymph node enlargement as a sign for oral cancer (86.4%). However, a large percentage of the participants in the clinical group failed to identify difficulty in swallowing (37.1%), tooth mobility and mucosal bleeding (49.8% for both) as signs and symptoms of oral cancer (Additional file 2).

Regarding the risk factors for oral cancer, the vast majority of participants in the clinical group correctly identified smoking (97.7%), HPV (88.2%), alcohol consumption (87.8%) and family history of oral cancer (76.0%) to be associated with the disease. However, a large percentage failed to identify sun exposure (53.8%) and severe anemia (68.3%) as possible risk factors for oral cancer. The clinical group showed significantly higher percentage of knowledge compared to the pre-clinical group (*p* < 0.001 for all comparisons, FET, Additional file 2).

### Oral cancer screening

**Fig. 1:**
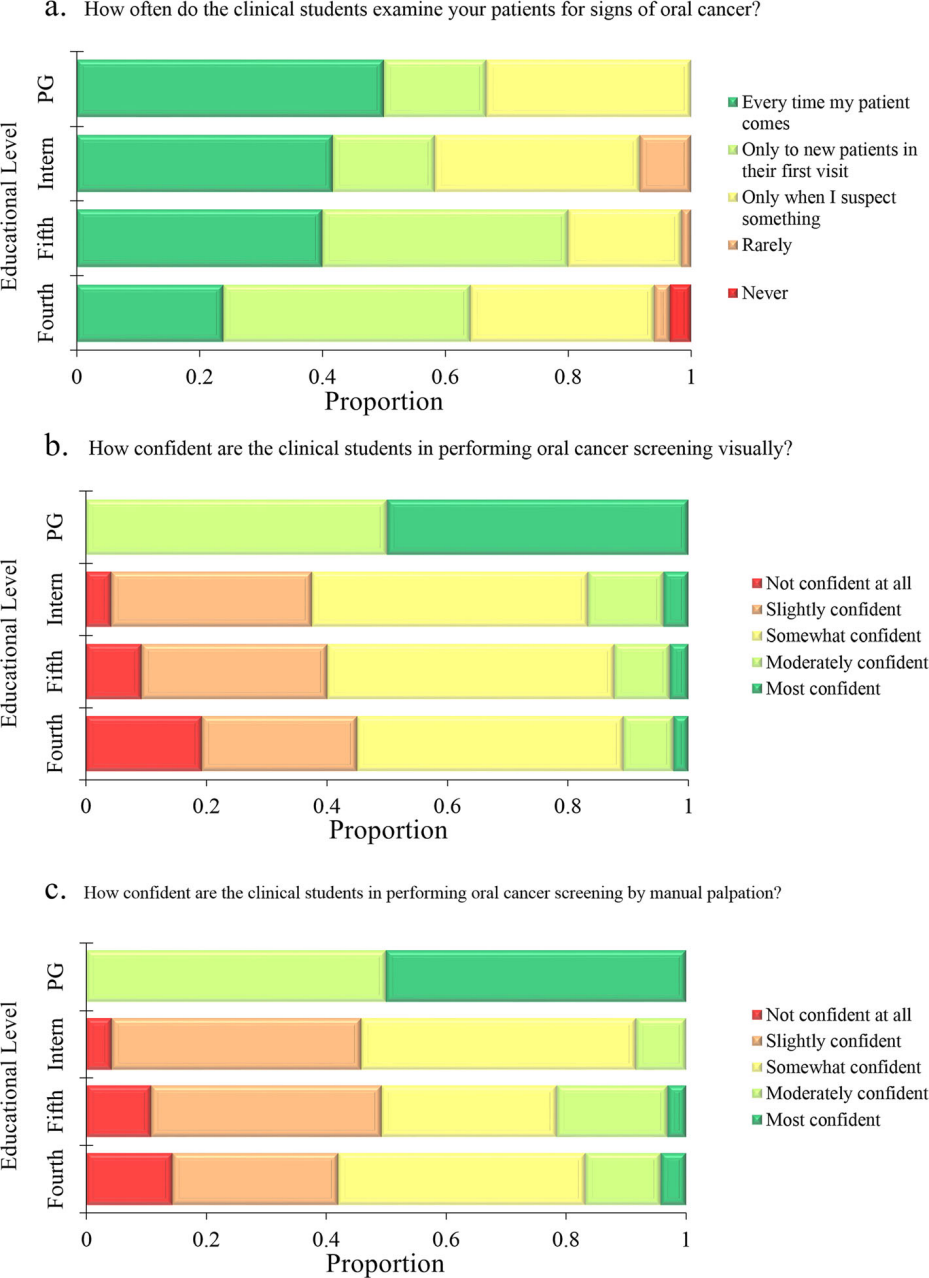
Attitude of clinical students at the University of Jordan towards screening of oral cancer. PG: Postgraduate maxillofacial residents. Fourth: 4th year doctor of dental surgery (DDS) students, Fifth: 5th year DDS students, PG: postgraduate maxillofacial residents

Regarding the frequency with which the participants in the clinical group examine patients for signs of oral cancer, the most common response was only to new patients in their first visit (*n* = 78, 36.8%). Slightly less than one-third of the participants in the clinical group reported that they will screen patients at every visit (*n* = 67, 31.6%), whereas more than a fourth of the participants in the clinical group reported that they only examine patients for signs of oral cancer if they suspect something (*n* = 57, 26.9%). Ten participants in the clinical group reported that they never or rarely examine their patients for signs of oral cancer (4.5%). Postgraduate residents were more likely to screen patients at every visit (50.0%) followed by interns (41.7%), 5th year students (40.0%) and 4th years students (23.9%), however this difference lacked statistical significance (*p* = 0.059, K-W, Fig. 1a).

Regarding the visual screening of oral cancer and on a scale from 1 to 5 (where 5 being the most confident), postgraduate residents showed the highest level of confidence (mean = 4.5, standard deviation [SD] = 0.55) followed by interns (mean = 2.79, SD = 0.88), 5th year clinical students (mean = 2.66, SD = 0.89) and lastly 4th year students (mean = 2.49, SD = 0.98, *p* < 0.001; K-W). Regarding manual palpation as a screening method for oral cancer and on a scale from 1 to 5 (where 5 being the most confident), postgraduate residents showed the highest level of confidence to perform screening (mean = 4.5, SD = 0.55), followed by 4th and 5th year clinical students (mean = 2.65, SD = 1.01 for both) and lastly interns (mean = 2.58, SD = 0.72, *p* = 0.002, K-W, Figs. 1b and c). No statistically significant differences were observed upon comparing gender and nationality groups regarding attitude towards screening (comparisons were done using t test).

The majority of clinical students reported that a reliable screening device for oral cancer is needed (*n* = 196, 92.9%) and that they need additional training on screening (*n* = 198, 93.4%).

### HPV knowledge

All participants in the clinical group who provided responses stated that they heard of HPV prior to participation in the survey (*n* = 214) compared to 65.0% (*n* = 52) of the participants in the pre-clinical group (*p* < 0.001, FET, Table 2).

A majority of the participants in the clinical group showed superior knowledge on HPV compared to the pre-clinical group participants, through providing correct responses to the following items: HPV can cause oral cancer (97.2% vs. 82.7%, *p* < 0.001), HPV can cause an STI (92.8% vs. 82.7%, *p* = 0.032) and that a person can have HPV without knowing it (91.0% vs. 75.5%, *p* = 0.004). In addition, participants in the clinical group showed better knowledge compared to the pre-clinical group participants in the following items: HPV causes AIDS (73.0% vs. 53.8%, *p* = 0.011) and HPV causes herpes and cold sores (56.9% vs. 24.5%, p < 0.001). We found no statistically significant difference in knowledge upon comparing the clinical and pre-clinical groups for the following items: Certain strains of HPV causes cervical cancer (94.8% vs. 88.7%, *p* = 0.121) and antibiotics can cure HPV infection (86.9% vs. 75.5%, *p* = 0.054). Both clinical and pre-clinical groups showed low level of knowledge to the following items without statistically significant difference: there is a vaccine for HPV (36.8% vs. 44.0%, *p* = 0.419) and most HPV infections resolve within a short period of time (22.1% vs. 32.0%, *p* = 0.145, FET for all comparisons, Additional file 2).

**Fig. 2:**
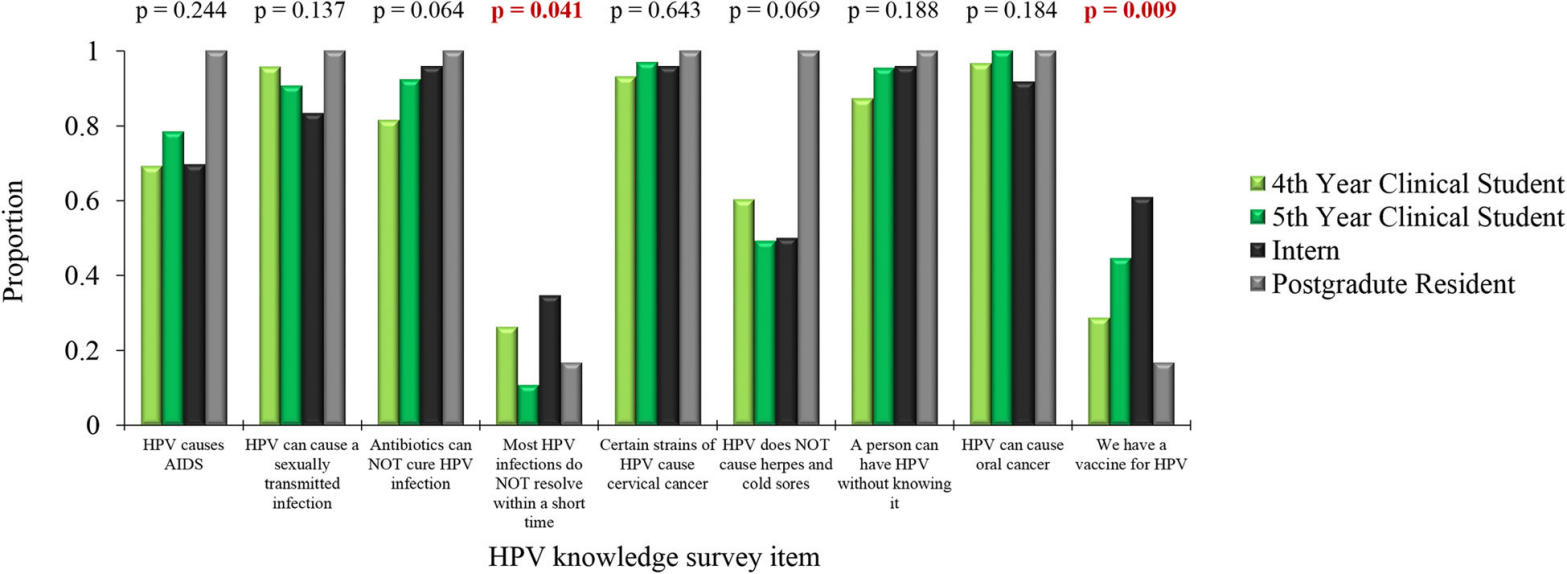
Human papillomavirus (HPV) knowledge among the clinical students at the University of Jordan. The survey items are stratified by level of education. *P* values were calculated using Kruskal-Wallis test. AIDS: acquired immunodeficiency syndrome. STI: sexually transmitted infection. Statistically significant values are shown in red

The stratification of the clinical group by level of education (4th year, 5th year, interns and residents) revealed statistically significant differences in HPV knowledge to the following items: there is a vaccine for HPV (28.8, 44.6, 60.9 and 16.7% correct responses among 4th year, 5th year, interns and residents respectively, *p* = 0.009, K-W) and most HPV infections resolve within a short period of time (26.3, 10.8, 34.8 and 16.7% correct responses among 4th year, 5th year, interns and residents respectively *p* = 0.041, K-W, Fig. 2).

The vast majority of the participants in the clinical group (97.2%) reported that it is important to enhance knowledge about HPV related oral cancer to the public. A majority of the participants in the clinical group (68.8%) stated that the best way to inform patients about HPV is to tell them that HPV can cause oral cancer compared to 31.2% who stated that the best way to inform patients about HPV is to tell them that the virus is associated with STI.

### Discussing personal topics with patients

**Fig. 3:**
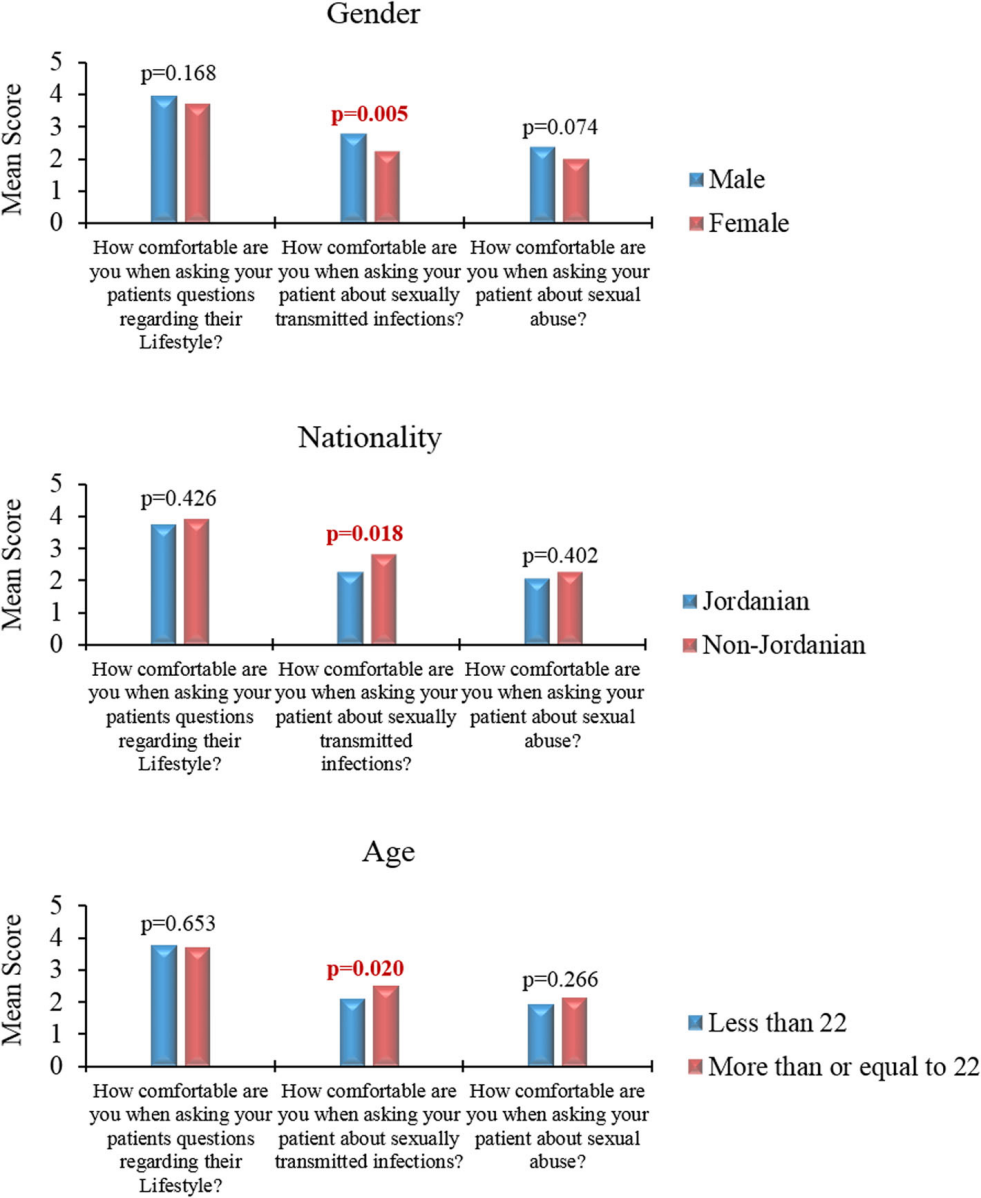
Attitude of clinical students at the University of Jordan towards discussing personal topics with patients. We scored the responses on the three items as follows: 1 = not comfortable at all, 2 = slightly comfortable, 3 = somewhat comfortable, 4 = moderately comfortable and 5 = most comfortable. The survey items are stratified based on gender, nationality and age. *P* values were calculated using the two-sided independent samples t-test

The responses were scored on the three items related to discussing personal topics with patients as follows: 1 = not comfortable at all, 2 = slightly comfortable, 3 = somewhat comfortable, 4 = moderately comfortable and 5 = most comfortable. Regardless of level of education, the majority of participants in the clinical group reported higher confidence in asking patients about their life style (mean = 3.74, SD = 1.19). However, confidence level decreased in relation to STI (mean = 2.34, SD = 1.24) and to sexual abuse (mean = 2.07, SD = 1.28). Stratified by gender, males were more comfortable than female participants to ask patients about STI (2.76 vs. 2.21, *p* = 0.005, t-test). Male participants had higher mean for the other two items but without statistical significance (Fig. 3).

In addition, Jordanian participants were more reluctant to ask about STI compared to non-Jordanian participants (mean = 2.26 vs. 2.81, *p* = 0.024, t-test). Moreover, older participants (more than or equal to 22 year (the median age of the clinical group) were more comfortable to ask about STI compared to younger participants (2.52 vs. 2.11, *p* = 0.016, t test, Fig. 3).

## Discussion

In the current study, the main objective was to assess knowledge and attitudes of dental students, interns and postgraduate maxillofacial surgery residents at UJ (the oldest and largest University in Jordan), towards HPV-related oral cancer. The UJ is one of two universities in the country that offers dentistry education. The current work was motivated by the accumulating evidence pointing to an increasing prevalence of high-risk HPV types as the underlying etiology for oral cancer worldwide [33–39]. In addition, HPV is anticipated to be the most common risk factor for oral cancer in the next decade [40]. Sufficient knowledge on oral cancer among general practitioners and dentists is critical, since early diagnosis is a decisive factor in reducing the morbidity and mortality from the disease [12, 41].

The assessment of level of knowledge and attitudes of the future dentists is crucial to highlight the limitations of the current curriculum taught locally. Moreover, the results of the study are expected to be beneficial at the country level, and might have an added value at the regional level, since a relatively large percentage of the participants were non-Jordanians and likely to practice dentistry in the neighbouring countries. The emphasis on dental staff awareness and the importance of dentists’ roles in the early diagnosis of oral cancer is invaluable particularly in the developing countries [42, 43].

Oral microbiology is an integral part of the dentistry curriculum. Certain microbes including HPV mandate special focus in dentistry not only in relation to cancer [44]. HPV represents an important pathogen causing oral lesions that are frequently encountered by the dentists, hence deep knowledge is important for the diagnosis and ensuring the patients about the nature of HPV-related oral disease [44].

The main findings of the study can be summarized as follows: First, the results revealed good overall knowledge among the clinical group regarding different aspects of oral cancer compared to pre-clinical group. The commonest anatomic sites, clinical manifestations and risk factors were correctly identified by the majority of the participants in the clinical group. However, a relatively large percentage of the clinical group failed to identify lips, buccal mucosa and palate as potential sites for oral cancer development. Lack of knowledge regarding buccal mucosa is particularly worrisome since these cancers generally have poor prognosis [5]. In addition, more than one-third of the participants in the clinical group failed to identify the frequent early lesions of oral cancer including the hard painless masses and mixed red-white lesions “erytholeukoplastic lesions” which might lead to delay in diagnosis that is associated with less-favourable outcome [45]. For the risk factors of oral cancer, the clinical group showed better results compared to the previous studies that were conducted in Jordan both among dental students and recently graduated medical and dental professionals [29, 30]. This might be related to intervention measures including improved dental education in light of the previous results of studies conducted in Jordan and globally. In addition, the rate of correct identification of smoking, alcohol consumption and HPV as risk factors for the disease was higher than the rates observed in some of the MENA countries among dentists and dental students, but in line with results of studies conducted in the Netherlands, Spain and Saudi Arabia [31, 32, 46–48].

Second, the participants in the clinical group showed varying attitudes toward oral cancer screening, with a stepwise increase in confidence to perform visual and manual palpation screening depending on the level of education. Improvements in the local educational curriculum are recommended including emphasizing the importance of conducting oral cancer screening as a routine practice.

Third, the participants in the clinical group showed better overall knowledge regarding HPV compared to participants in the pre-clinical group. Nevertheless, certain gaps were identified concerning basic knowledge of the virus. Despite the fact that oral cancer represents the most ominous outcome for HPV infection in the oral cavity, HPV comprises more than 200 types, with varying oncogenic potential [49]. Knowledge of these types and their associated clinical diseases is indispensable for dentists since HPV infections are common in the oral cavity and can manifest in different ways [44].

Fourth, participants in the clinical group were reluctant to discuss issues related to STI and history of sexual abuse with patients. This was particularly evident among female Jordanian participants. The role of communication with patients to reveal history of STIs and sexual abuse is important since HPV types that are associated with oral cancer are usually transmitted through oral sex [50–52]. The aforementioned reluctance might be related to cultural and religious attitude towards discussing these issues in the MENA. However, similar difficulties in discussing these topics were also reported among American dentists and Spanish dental students [26, 32].

Finally, the majority of participants aspire for more training and devices that can aid in oral cancer screening which should be addressed. Possible options include continued education in the form of workshops, practical training sessions and awareness campaigns.

The current study had certain limitations as follows: One limitation inherent in a large number of surveys is the possibility that a fraction of participants responded in a way they believe to be suitable for the authors conducting the survey. During the distribution of the questionnaires to participants, it was attempted to minimize this limitation by declining to answer questions or responding to enquiries regarding the different questionnaire items. Sampling error is another potential limitation as the choice of participants to take part in the study might have been influenced by their previous knowledge on oral cancer and HPV which was evident by the low percentage of the pre-clinical participants who agreed to take part in the study. Non-response error should also be considered in spite of our attempt to formulate clear questions that were assessed and modified using the pilot testing. To reduce coverage error we tried to sample as much students as possible during the narrowest feasible period. Another potential limitation was the female predominance in the sample which might have influence on some results particularly those related to discussion of personal topics with patients. Since the study was conducted in a single institution (UJ), our results might not reflect the overall knowledge and attitudes of dental students in the country. Finally, the higher response rate among fourth year clinical students might be explained by the participation of three authors who are themselves at the same educational level, which might motivated some reluctant respondents to participate.

## Conclusions

The majority of the participants in the clinical group at UJ showed better knowledge regarding oral cancer in various survey items compared to the pre-clinical group participants. Nevertheless, gaps in knowledge were observed particularly in aspects related to clinical presentation of the disease which might be an impediment to life-saving intervention measures. The overall knowledge of the participants in the clinical group regarding HPV was satisfactory, albeit with gaps in certain aspects (e.g. availability of vaccine). Participants in the clinical group showed reluctance to discuss topics like STIs and history of sexual abuse with the patients (particularly among female participants). This issue can be addressed through improved educational training programs. Knowledge about HPV-related oral cancer is crucial and is recommended to be taught as an integral part of the basic curriculum and clinical training of dental students. A follow-up comparative study is recommended among medical students.

## Additional files

Additional file 1: The paper-based questionnaire template that was used in the study. (PDF 115 kb)

Additional file 2: Additional results. (PDF 64 kb)

## Data Availability

The paper-based questionnaire form is provided in Additional file 1. Additional results are presented in Additional file 2.

## Abbreviations

DDS: Doctor of dental surgery
FET: Two-sided Fisher’s exact test
HPV: Human papillomavirus
IQR: Interquartile range
JUH: Jordan University Hospital
K-W: Kruskal-Wallis test
MENA: Middle East and North Africa
M-W: Mann-Whitney *U* test
SD: Standard deviation
STI: Sexually transmitted infection
UJ: University of Jordan
