# Supplementary material for: Dental students’ awareness and attitudes toward HPV-related oral cancer: a cross sectional study at the University of Jordan
Source: BMC Oral Health. 2019 Aug 1;19:171. doi: 10.1186/s12903-019-0864-8 (PMC6670240; doi:10.1186/s12903-019-0864-8)
Supplement: Supplementary file 1 — The paper-based questionnaire template that was used in the study. (PDF 115 kb) [file 12903_2019_864_MOESM1_ESM.pdf]

Thank you very much for agreeing to participate in this survey.

This project aims to assess the awareness of dental students and postgraduate dental professionals on oral cancer. The research project intends to evaluate the ability of dental professionals to examine and diagnose premalignant lesions. It also intends to see if there is a difference in the results between the undergraduate and postgraduate dentists. Our objective is that the research findings will contribute to the development of a framework designed to fill in the gap where students and/or the dentists lack.

The information provided by you in this questionnaire will be used for research purposes. It will not be used in a manner which would allow identification of your individual responses.

Section A

Age: ……………

Gender:

☐ Male

☐ Female

Nationality: ……………

Level of education:

☐ 2nd year

☐ 3rd year

☐ 4th year

☐ 5th year

☐ Intern

☐ Postgraduate resident

Section B. Oral cancer Knowledge: (You can tick more than one box)

1- Have you ever heard of oral cancer?

☐ Yes

☐ No (If no, then thank you for participating and no need to proceed with filling the questionnaire).

2- **Where do you think oral cancer is found in the oral cavity?**

- ☐ Tongue
- ☐ Lips
- ☐ Palate
- ☐ Jaw bone
- ☐ Buccal mucosa
- ☐ Floor of the mouth
- ☐ Other sites: ……………

3- **Which of the following describes the clinical appearance of the early lesion of oral cancer?**

- ☐ White lesion
- ☐ Red lesion
- ☐ Ulcer
- ☐ Mass
- ☐ Others: ……………

4- **What are the signs and symptoms of oral cancer?**

- ☐ Mucosal bleeding
- ☐ Difficulty in swallowing
- ☐ Tooth mobility
- ☐ Lymph node enlargement
- ☐ Others: ……………

5- **What are the risk factors for oral cancer?**

- ☐ Smoking
- ☐ Alcohol
- ☐ Positive family history
- ☐ HPV
- ☐ Severe anemia
- ☐ Sun exposure

Section C. Screening:

These questions are for clinical students, interns and postgraduate residents.

1. How often do you examine your patients for signs of oral cancer?	☐ Every time my patient comes	☐ Only to new patients in their first visit	☐ Only when I suspect something	☐ Rarely	☐ Never
2. How confident are you in performing oral cancer screening visually? (1-5) 5 being the most confident	☐ 1	☐ 2	☐ 3	☐ 4	☐ 5
3. How confident are you in performing oral cancer screening by manual palpation? (1-5) 5 being the most confident	☐ 1	☐ 2	☐ 3	☐ 4	☐ 5
4. Would you like reliable screening device for oral cancer?				☐ Yes	☐ No
5. Do you think you need additional training in oral cancer screening?				☐ Yes	☐ No

Section D. HPV knowledge:

1. Have you ever heard of HPV?	☐ Yes	☐ No
2. HPV causes AIDS	☐ True	☐ False
3. HPV can cause a sexually transmitted infection	☐ True	☐ False
4. Antibiotics can cure HPV infection	☐ True	☐ False
5. Most HPV infections resolve within a short time	☐ True	☐ False
6. Certain strains of HPV causes cervical cancer	☐ True	☐ False
7. HPV causes herpes and cold sores	☐ True	☐ False
8. A person can have HPV without knowing it	☐ True	☐ False
9. HPV can cause oral cancer	☐ True	☐ False
10. We have a vaccine for HPV	☐ True	☐ False
11. Do you think it is important to enhance knowledge about HPV related oral cancer to public?	☐ Yes	☐ No
12. What do you think is the best way to inform the patient about HPV?	☐ Tell them about the risk for oral cancer	☐ Tell them HPV is an infectious disease

Section E. Discussing personal topics:

Please answer these questions with 5 being very comfortable and 1 being the least comfortable

1. How comfortable are you when asking your patients questions regarding their Lifestyle?	☐ 1	☐ 2	☐ 3	☐ 4	☐ 5
2. How comfortable are you when asking your patient about sexually transmitted infections?	☐ 1	☐ 2	☐ 3	☐ 4	☐ 5
3. How comfortable are you when asking your patient about sexual abuse?	☐ 1	☐ 2	☐ 3	☐ 4	☐ 5

Thank You
