# Supplementary material for: Dental students’ awareness and attitudes toward HPV-related oral cancer: a cross sectional study at the University of Jordan
Source: BMC Oral Health. 2019 Aug 1;19:171. doi: 10.1186/s12903-019-0864-8 (PMC6670240; doi:10.1186/s12903-019-0864-8)
Supplement: Supplementary file 2 — Additional results. (PDF 64 kb) [file 12903_2019_864_MOESM2_ESM.pdf]

Dental students' awareness and attitudes toward HPV-related oral cancer: a cross sectional study at the University of Jordan. Sallam M. *et al*.

Supplementary File 2

Additional Results

Oral cancer knowledge

Other potential sites for oral cancer reported by the participants included salivary glands, gingivae, pharynx and larynx. No statistical differences were observed upon comparing the gender and nationality in the clinical group, except for the buccal mucosa where it was identified correctly among 69.2% of the Jordanian participants as opposed to 43.6% among non-Jordanian participants (p=0.005, FET), and the jaw bone where it was identified correctly among 66.2% of the Jordanian participants as opposed to 43.6% among non-Jordanian participants (p=0.015, FET).

Regarding possible oral cancer early signs, no statistical difference was observed upon comparing the gender and nationality groups. Other early signs reported by the clinical group included painless mass and non-healing lesions (n=7).

For the clinical manifestations of oral cancer, no statistical differences were observed upon comparing the gender and nationality groups. Other clinical manifestations reported by the participants in the clinical group included painless mass and tooth root resorption (n=6).

Regarding the risk factors for oral cancer, No statistical differences were observed upon comparing the gender and nationality groups, except for the family history which was identified correctly among 82.0% of the Jordanian participants as opposed to 61.5% among non-Jordanian participants (p=0.015, FET), and severe anemia which was identified correctly

among 34.6% of the Jordanian participants as opposed to 15.4% among non-Jordanian participants (p=0.028, FET).

HPV knowledge

Gender- and nationality-based comparisons for all HPV knowledge-related items revealed no statistically significant difference for both the whole group of participants and among clinical group.
